# Complete genome sequence of a *Pasteurella multocida* isolate from a pig in Australia

**DOI:** 10.1128/mra.00241-24

**Published:** 2024-07-30

**Authors:** David Chau, Patrick J. Blackall, Conny Turni, Lida Omaleki

**Affiliations:** ^1^Queensland Alliance for Agriculture and Food Innovation, University of Queensland, St. Lucia, Queensland, Australia; Loyola University Chicago, Chicago, Illinois, USA

**Keywords:** *Pasteurella multocida*, complete genome sequence, antibiotic resistance, beta-lactamases

## Abstract

Here, we announce the complete genome sequence of a *Pasteurella multocida* isolate (PM1463) obtained from a diseased pig as a part of routine diagnostic investigations by the field veterinarian in Queensland, Australia. The assembly consists of a 2,321,605-bp chromosome and a 4,769-bp plasmid. The assembly has an average GC content of 40.33%.

## ANNOUNCEMENT

PM1463 was isolated from a sick pig by front-line diagnostic laboratories in 2011 and forwarded to the Microbiology Research Group for confirmation and typing, where it was stored in a −80°C freezer. The isolate was identified as *Pasteurella multocida* of lipopolysaccharide type 1 by multiplex PCR (1), shown to be resistant to ampicillin and penicillin (2), to carry *bla*_ROB-1_ coding for β-lactam resistance (3), to belong to the multilocus sequence type ST 11 based on the Rural Industries Research and Development Corporation (RIRDC) scheme (4) and to be Heddleston serovar 4 (1). While the complete genome of an ST394 bovine *P*. *multocida* isolate (5) and several ST20 avian *P. multocida* isolates (6) from Australia have been recently deposited to public databases, complete genome of an Australian porcine *P. multocida* isolate have not been reported previously.

The isolate was revived on sheep blood agar and incubated overnight at 37°C in aerobic condition for DNA extraction. DNA was extracted using the Puregene Tissue Core kit (QIAGEN), according to the manufacturer’s instructions. The extracted DNA was sent to the Australian Centre for Ecogenomics Sequencing Service at the University of Queensland (St. Lucia, QLD 4072, Australia) where a Nextera DNA Flex kit (Illumina, San Diego, CA, USA) was used for library preparation according to the manufacturer’s protocol. The DNA libraries were then sequenced on an Illumina NovaSeq 6000 platform producing paired-end (150 bp) reads. Fastp v0.20.1 (7) was used for trimming the raw Illumina reads with parameters of 20-bp front and 5-bp tail trimming. The quality of the reads was then accessed by FastQC v0.74 (8).

**Fig 1:**
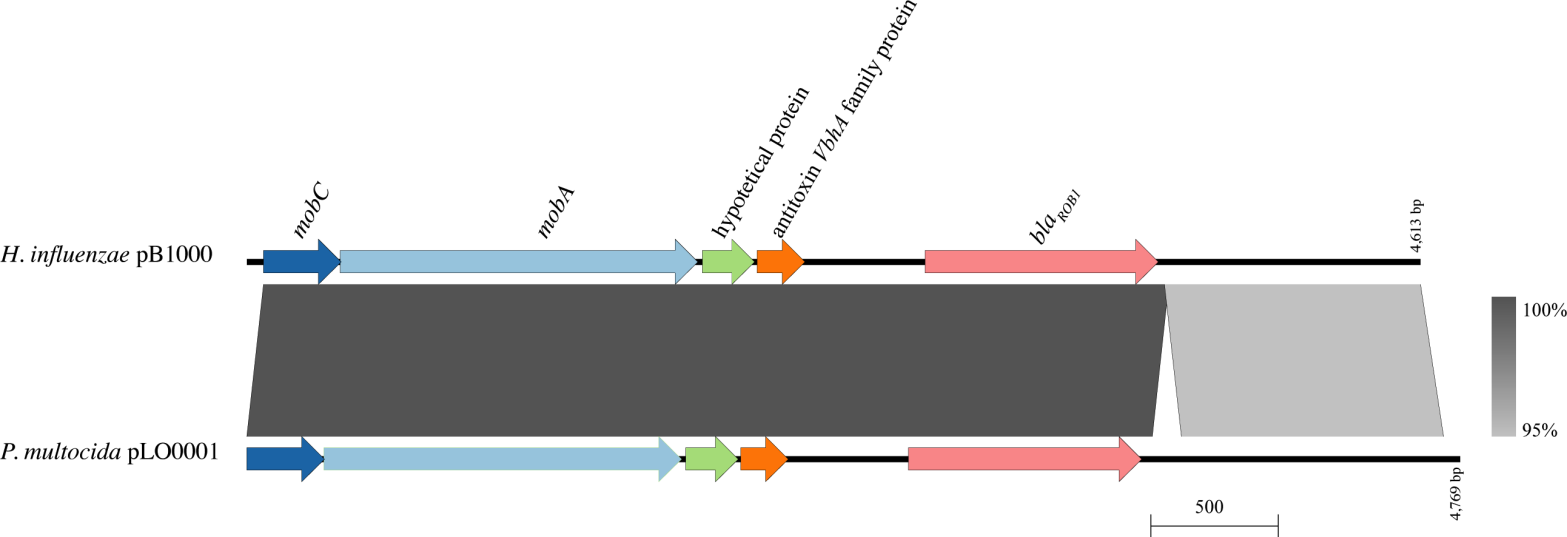
Comparison of the plasmid carried by PH1463 (bottom) and pB1000 (top) carried by human pathogen *H. influenzae*. Arrows correspond to genes and are labeled on the top. BLASTn comparison was produced using EasyFig v2.2.2 (19).

An aliquot of the same DNA extract was also used for library preparation using the Oxford Nanopore Technology (ONT) ligation sequencing kit (SQK-LSK109) and then sequenced on an ONT flow cell type R9.4.1 (FLO-MIN109). DNA was not sheared and not size selected before sequencing. Guppy v6.2.1 (9) with a high-accuracy model was used for base calling the raw ONT reads. The base-called reads were filtered and the quality assessed using Nanofilt and NanoStat embedded in nanopack v.1.1.0 (10) (Table 1), with parameters of a minimum quality score of 10, a minimum length of 150 bp, and a 75-bp head trim and 25-bp tail trim. After this, different bioinformatic tools were accessed and used in the Galaxy Australia platform (with the default parameter unless otherwise specified). The complete genome of strain PM1463 was subjected to *de novo* assembly with Trycycler v0.5.4 (11). Two contigs were generated as a result of the assembly with both of them successfully circularized. Racon v1.5.0 (12) was used for error correction of the contigs using the Illumina reads. The lengths of the two circular contigs were 2,321,605 and 4,769 bp (Table 1). The quality assessment was performed with Quast v5.2.0 (13) and BUSCO v5.5.0 (14) with the *Pasteurellales* lineage data set. The results from BUSCO indicated 1,099 genes to be “Complete,” and one gene was “Missing” out of 1,100 ortholog groups. The genome was annotated via the National Center for Biotechnology Information Genome Annotation Pipeline (PGAP) v6.6 (15, 16). The MLST and antimicrobial resistance genes were identified with statamr Galaxy Version 0.10.1 (17). The RIRDC-MLST was confirmed as 11, where the MLST profile is *adk*-2, *est*-9, *pmi*-7, *zwf*-8, *mdh*-7, *gdh-*3, and *pgi-*9. The genome was searched for potential plasmids with MOB-suite v3.0.3 (18). The smaller contig was identified as a plasmid with a mash neighbor distance of 0.00294054 with plasmid PB1000 previously identified in *Haemophilus influenzae* (19) (Accession number NC014813). Alignment of the two plasmids using Clustal Omega indicated 95% nucleotide identity. Blastn comparison was produced using EasyFig v2.1 (20), which is shown in Fig. 1.

**TABLE 1:** Statistic for the sequencing and the assembly

Parameter	Data
Sequence reads statistics	
Illumina paired-end read length (nt)	150
No. of Illumina left read (R1) (raw)	5,301,532
No. of Illumina right read (R2) (raw)	5,301,532
No. of Illumina left read (R1) (filtered)	3,881,516
No. of Illumina right read (R2) (filtered)	3,881,516
No. of Nanopore reads (raw)	50,000
No. of Nanopore bases (raw)	385,120,530
*N*_50_ of Nanopore reads (raw)	20,581
No. of Nanopore reads (filtered)	44,940
No. of Nanopore bases (filtered)	353,161,377
*N*_*50*_ of Nanopore reads (filtered)	20,708
Assembly statistics	
No. of contigs	2
Contig 1 size	2,321,605
Contig 1 coverage	148^*a*^
Contig 2 size	4,769
Contig 2 coverage	98^*a*^
Total length of genome (bp)	2,326,374
GC content (%)	40.33
*N*­­_50_	2,321,605

^*a*^ Estimated with long read assembly.

## Data Availability

The genome sequence of *P. multocida* PM1463 is available in NCBI under BioProject PRJNA1071487, accession number GCA_037021625.1 (CP144454 and CP144455 for the chromosome and plasmid, respectively). Raw reads are available as BioSample SAMN39849450 with Illumina reads available via SRA SRX19395327 and the reads from the ONT sequencing through accession number SRR27896389.
